# Situations predisposing primary care patients to use antibiotics without a prescription in the United States

**DOI:** 10.1017/ash.2024.361

**Published:** 2024-09-09

**Authors:** Lindsey A. Laytner, Barbara W. Trautner, Susan Nash, Fabrizia Faustinella, Roger Zoorob, Kiara Olmeda, Michael K. Paasche-Orlow, Larissa Grigoryan

**Affiliations:** 1 Department of Family and Community Medicine, Baylor College of Medicine, Houston, TX, USA; 2 Center for Innovations in Quality, Effectiveness, and Safety (IQuESt), Michael E. DeBakey Veterans Affairs Medical Center, Houston, TX, USA; 3 Department of Medicine, Section of Health Services Research, Baylor College of Medicine, Houston, TX, USA; 4 Department of Medicine, Tufts Medical Center Boston, MA, USA

## Abstract

**Background:**

Patients’ situations can impact their intentions to use antibiotics without medical guidance (non-prescription use) in the future. This survey determines the prevalence of intended (future) use of non-prescription antibiotics for 13 predefined situations and identifies the sociodemographic characteristics associated with intended use for these types of situations.

**Methods:**

Patient surveys (N = 564) were conducted from January 2020 to June 2021 in the waiting rooms of 6 safety-net primary care clinics and 2 emergency departments in a private healthcare system. We used principal component analysis to identify 3 situational summary factors: barriers to a doctor visit, accessibility of non-prescription antibiotics, and previous symptom relief with antibiotics. Multivariate linear regression identified the sociodemographic predictors associated with each summary factor.

**Results:**

The most common situations triggering patients to use non-prescription antibiotics were a perceived high cost of doctor visits (29.8%), having leftover prescription antibiotics (50.4%), and experiencing symptom relief with prior use of antibiotics (47.5%). Multivariate regression results revealed that younger patients (*P* < 0.04) and patients attending the safety-net health system (*P* < 0.001) had more intended use of non-prescription antibiotics for all 3 summary factors.

**Conclusions:**

Future stewardship interventions should consider the types of situations that drive patients’ decisions to use antibiotics without a prescription. Interventions aimed at reducing barriers to health care (eg, high costs and long waits associated with doctor appointments) and educating individuals on medically appropriate, nonantibiotic treatment options may reduce antibiotic use and antimicrobial resistance.

## Introduction

Reducing inappropriate antibiotic use in the outpatient setting is a global health priority and a core objective of the 2020–2025 US National Action Plan for Combating Antibiotic-Resistant Bacteria.^1–5^ Using antibiotics without medical guidance (non-prescription antibiotic use) can lead to unnecessary use of antibiotics for viral infections and likewise is a threat to patient safety and public health by increasing patients’ risk of adverse effects, superinfections, and disruption of the microbiome and may promote the development of antimicrobial resistance.^1–4^

Non-prescription antibiotic use is prevalent in both low- and high-income countries.^3,6–9^ Recent studies have shown that non-prescription antibiotic use is prevalent in the United States, ranging from 20% to 45%, depending on the patient populations and community groups surveyed.^10–12^ Non-prescription antibiotics were obtained from leftover/previously prescribed courses, friends/relatives and social networks, stores and markets in the United States (illegally sold), over-the-counter sales in other countries, and Internet sources.^10,13,14^

Previous studies have found that antibiotic misuse is influenced by patient and healthcare system determinants.^9,10,13,14^ Patient-level factors include younger age, lower education levels, using antibiotics in the past, and storing antibiotics in their homes.^9,10,13,14^ Healthcare system factors included having poor access to health care (eg, high healthcare costs, transportation problems) or difficulties with medical visits (eg, long clinic waits to be seen by the clinician or to get an appointment).^13,14^ However, most of the US-based studies on the determinants of non-prescription antibiotic use have focused on Hispanic and Latinx immigrant populations with low income and education, minimal to no health insurance coverage, and limited English language proficiency.^12,15–17^

Large-scale surveys investigating the independent effects of social factors on patients’ decisions to use antibiotics without a prescription are lacking in socioeconomically and linguistically diverse US-based primary care settings. Our study aimed to (1) determine the prevalence of intended (future) use of non-prescription antibiotics for 13 predefined situations and (2) identify the social and demographic predictors associated with intended use for these types of situations in diverse outpatient settings.

## Study methods

### Participants

#### Design

A cross-sectional survey on non-prescription antibiotic use was conducted in Texas between January 2020 and June 2021.^10^ Surveys were administered by trained research coordinators in the waiting rooms of 6 public, safety-net primary care clinics (eg, 3 continuity and 3 same-day/walk-in) and 2 private emergency departments (EDs) that serve racially, ethnically, and socioeconomically diverse patients.^10^

#### Recruitment

Clinic personnel provided recruitment flyers to each participant who checked in for a primary care visit.^10^ Interested adult patients volunteered to be surveyed anonymously. Individuals under 18 years old or adults who were unable to answer the survey questions were excluded.^10^ All patients were offered a $15 incentive for their time.^10^

### Survey

The survey interviews (Appendix 1) were conducted in person (when permitted during the pandemic) or remotely (via teleconferencing) in the patients’ preferred language (English or Spanish). Additional survey details (eg, design, recruitment, and survey guide development) are published elsewhere.^10^

### Survey variables

To assess patients’ endorsement of non-prescription antibiotics (intended use) in various situations, we queried, “If you were feeling sick, would you take antibiotics in the following situations without contacting a doctor/nurse/dentist/clinic?” Each patient was then presented with 13 predefined items/situations,^18^ ranging from “You have leftover antibiotics at home from a previous prescription” to “Antibiotics are cheaper than over-the-counter cold and flu medications” (Appendix 1). Each situation/item was discussed and iteratively designed with input from the clinical staff, a consultant in linguistic and cultural competency for Hispanic communities, a health literacy expert, and our patient advisory board at the safety-net clinics to ensure applicability to this patient population.^10^ Patients could respond with “yes,” “no,” or “I don’t know” after each situation was presented to them. Individuals who reported “yes” to any of the situations were scored as endorsing non-prescription antibiotic use.

Patients’ sociodemographic factors were also assessed (Table 1). These sociodemographic factors included patients’ age, gender, race and ethnicity, education level, health insurance status, healthcare system, health literacy, and language preference. Race and ethnicity categories included non-Hispanic Black/African American, Hispanic or Latinx, non-Hispanic White, and other. Education levels included less than high school, high school or GED, or some college and above. Health insurance status included private or Medicare, public or Medicaid or county financial assistance program (CFAP), and self-pay. The CFAP includes those patients who have benefits from Harris County, which allows access to public clinic providers at either very low or no cost. Health literacy was assessed using a brief screening tool by Chew and colleagues.^19,20^

**Table 1.** Patient sociodemographic characteristics by healthcare system. Differences between the public safety-net and private healthcare systems (*P* < 0.05) are significant

	All healthcare systems (N = 564)	Public safety-net clinics (N = 409)	Private EDs (N = 155)	Difference between the healthcare systems
Patient characteristics	n (%)	n (%)	n (%)	*P* value^a^
Median age [range]	50 [19–92]	53 [19–77]	41 [19–92]	***P*** **< 0.001**
Gender/sex				*P* = 0.181
Female	407 (72)	302 (74)	105 (68)	
Male	157 (28)	107 (26)	49 (32)	
Race and ethnicity				***P*** **< 0.001**
Non-Hispanic Black/African American	186 (33)	144 (35)	42 (27)	
Hispanic or Latinx	263 (47)	222 (54)	41 (26)	
Non-Hispanic White	89 (16)	32 (8)	57 (37)	
Other^b^	26 (4)	11 (3)	15 (10)	
Education				***P*** **< 0.001**
Less than high school	92 (16)	82 (20)	10 (7)	
High school or GED	225 (40)	184 (45)	41 (26)	
Some college and above	247 (44)	143 (35)	104 (67)	
Health insurance status				***P*** **< 0.001**
Private or Medicare	207 (37)	94 (23)	113 (73)	
Medicaid or county financial assistance program^c^	319 (56)	308 (75)	11 (7)	
Self-pay	38 (7)	7 (2)	31 (20)	
Healthcare system				***P*** **< 0.001**
Private (*ED*)	155 (27)	--		
Public safety-net	409 (73)		--	
*Same day*	--	191 (47)	--	
*Health center*	--	218 (53)	--	
Survey language				***P*** **< 0.001**
Spanish	155 (27)	143 (35)	12 (8)	
English	409 (73)	266 (65)	143 (92)	
Health literacy^d^				*P* = 0.257
Adequate health literacy	391 (69)	278 (68)	113 (73)	
Inadequate health literacy	173 (31)	131 (32)	42 (27)	

a Boldface indicates statistical significance (*P* value <0.05).

b Other includes mixed race (Black/African American and Hispanic or “Afro-Latin”) and Asian.

c The county financial assistance program includes those patients who have benefits from a county, which allows access to public clinic providers at either very low or no cost.

d Individuals endorsing (answering “yes”) having difficulty understanding written information, confidence in filling out medical forms by themselves, and someone helps them read clinic or hospital materials.^19,20^

### Ethics

This study was approved by the Institutional Review Board for Baylor College of Medicine and Affiliated Hospitals (protocol H-45709).

### Statistical methods

#### Comparison of prevalence of intended use between safety-net clinics and private EDs

χ^2^ tests for categorical variables were used to determine if there were any significant differences among 13 situations between the private and public safety-net healthcare systems. A *P* value of <0.05 was considered significant (Table 2).

**Table 2.** Patient-reported intended use for each situation by the healthcare system

	Overall (N = 564)	Safety-net clinics (N = 409)	Private EDs (N = 155)	Difference between safety-net and EDs
Patient characteristics	n (%)	n (%)	n (%)	*P* value^a^
*If you were feeling sick, would you take antibiotics in the following situations without contacting a doctor/nurse/dentist/clinic?* (Agree)				
Summary factor 1: Barriers to a doctor’s visit				
You cannot take time off work.	146 (25.9)	126 (30.8)	20 (12.9)	**<.001**
You have no time to go to the doctor because of family responsibilities.	149 (26.4)	126 (30.8)	23 (14.8)	**<.001**
You cannot get to the doctor’s office because of transportation problems.	144 (25.5)	128 (31.3)	16 (10.3)	**<.001**
The doctor’s office hours are not convenient for you.	150 (26.6)	135 (33)	15 (9.7)	**<.001**
The doctor has no time to see you when you are sick.	154 (27.3)	138 (33.7)	16 (10.3)	**<.001**
A visit with a doctor is too expensive.	168 (29.8)	137 (33.5)	31 (20)	**0.007**
Summary factor 2: Accessibility of non-prescribed antibiotics				
You have leftover antibiotics at home from a previous prescription.	284 (50.4)	227 (55.5)	57 (36.8)	**<.001**
Friends/relatives give you antibiotics.	126 (22.3)	106 (25.9)	20 (12.9)	**0.003**
You can buy antibiotics without a prescription in the United States.	108 (19.1)	88 (21.5)	20 (12.9)	**0.018**
You can buy antibiotics without a prescription in another country.	101 (17.9)	79 (19.3)	22 (14.2)	**0.044**
Antibiotics are cheaper than over-the-counter cold and flu medications.	93 (16.5)	78 (19.1)	15 (9.7)	**0.027**
Summary factor 3: Previous symptom relief with antibiotics				
You got better by taking this antibiotic before.	268 (47.5)	210 (51.3)	58 (37.4)	**0.012**
Your doctor prescribed you this antibiotic for the same symptoms before.	280 (49.6)	220 (53.8)	60 (38.7)	**<.001**

a Boldface indicates statistical significance (*P* value <0.05).

#### Situational summary factors scores

We used principal components analysis (PCA) to demonstrate the dimensionality of the 13 situations (items). We used PCA as a data reduction technique and confirmed the factor structure underlying these situations, identifying distinct “summary factors.” Smaller composite summary factors were created for items with high correlation (item factor loading >0.6) (Table 3). Three distinct situational summary factors (consistent with the conceptual basis) emerged from the 13 situations/items entered, including (1) barriers to a doctor’s visit (Cronbach’s α = 0.96; mean inter-item correlation [IC] = 0.79), (2) accessibility of non-prescription antibiotics (Cronbach’s α = 0.81; mean IC = 0.48), and (3) previous symptom relief with antibiotics (Cronbach’s α = 0.95; mean IC = 0.9) (Table 3).

**Table 3.** Dimensionality of the 13 situations/items, including item factor loadings and reliability statistics per situational summary factor

Situational summary factors and situations/items^a^	Situation/item factor loading^b^		
	1	2	3
Summary factor 1: Barriers to a doctor’s visit			
You cannot take time off work.	0.92		
You have no time to go to the doctor because of family responsibilities.	0.94		
You cannot get to the doctor’s office because of transportation problems.	0.93		
The doctor’s office hours are not convenient for you.	0.94		
The doctor has no time to see you when you are sick.	0.93		
A visit with a doctor is too expensive.	0.80		
Summary factor 2: Accessibility of non-prescribed antibiotics			
You have leftover antibiotics at home from a previous prescription.		0.61	
Friends/relatives give you antibiotics.		0.83	
You can buy antibiotics without a prescription in the United States.		0.85	
You can buy antibiotics without a prescription in another country.		0.80	
Antibiotics are cheaper than over-the-counter cold and flu medications.		0.72	
Summary factor 3: Previous symptom relief with antibiotics			
You got better by taking this antibiotic before.			0.97
Your doctor prescribed you this antibiotic for the same symptoms before.			0.97
Cronbach alpha	α = 0.96	α = 0.81	α = 0.95
Mean inter-item correlation	0.79	0.48	0.90

a Patients could respond to each situation with “yes,” “no,” or “I don’t know.” Individuals who reported “yes” endorsed intended non-prescription antibiotic use, and those responding with “no” or “I don’t know” were categorized as non-endorsers of intended non-prescription antibiotic use.

b Loading scores >0.6 were included in the summary factor score.

Each patient was assigned a composite score for each situational summary factor by using the sum of all “yes” responses to each of the situations/items per summary factor. Scores >0 indicate that the patient answered yes to one or more situations within each summary factor. For example, a patient with a score of 0 for summary factor 1 would indicate that they did not experience any barriers to a doctor’s visit (Table 3). Furthermore, each situational summary factor and the patients’ composite scoring were determined as described below.

***Summary factor 1. “Barriers to a doctor visit”*** included 6 items relating to the patients’ (1) work responsibilities, (2) family responsibilities, (3) access to transportation, (4) ability to get a convenient appointment time, (5) difficulty getting an appointment with the doctor when sick, and (6) the perceived cost of the visit (Table 3). Each patient was assessed on a scale of 0–6. For example, a patient’s summary factor 1 composite score of 0 indicated they did not answer “yes” to any situations/items related to the barriers to a doctor visit, and 6 indicated they responded “yes” to all the situations/items presented (Table 4).

**Table 4.** Range of patients’ composite summary scores for each situational summary factor

Situational indices	N	N (%) Respondents with 1 or more situations reported	N (%) Respondents without any situations reported	Min.	Max.
Summary factor 1: Barriers to a doctor’s visit	523	192 (37)	331 (63)	0	6
Summary factor 2: Accessibility of non-prescribed antibiotics	519	279 (54)	240 (46)	0	5
Summary factor 3: Previous symptom relief with antibiotics	547	284 (53)	263 (48)	0	2

***Summary factor 2. “Accessibility of non-prescription antibiotics”*** consisted of 5 items relating to patients’ having (1) leftover antibiotics in their possession, (2) non-prescribed antibiotics given to them by friends or relatives, (3) purchased antibiotics without a prescription in the United States, (4) purchased antibiotics abroad, and (5) beliefs that antibiotics are less expensive than over-the-counter cold and flu medications (Table 3). Each patient was assessed on a scale of 0–5. For example, a patient’s summary factor 2 composite score of 0 indicated they did not answer “yes” to any situations/items related to the accessibility of non-prescription antibiotics, and 5 indicated they responded “yes” to all the situations/items presented (Table 4).

***Summary factor 3. “Previous symptom relief with antibiotics”*** included two items: (1) the patient got better by taking an antibiotic in the past, and (2) the doctor prescribed the patient an antibiotic in the past for the same or similar symptoms (Table 3). Each patient was assessed on a scale of 0–2. For example, a patient’s summary factor 3 composite score of 0 indicated they did not answer “yes” to any situations/items related to previous symptom relief with antibiotics, and 2 indicated they responded “yes” to all the situations/items presented (Table 4).

### Linear regression models

Multivariate linear regressions were performed for each summary factor (outcome) to determine the patient-level factors associated with the situations that influence patients’ endorsement of non-prescription use. Predictors for each model included sociodemographic variables that were significant in univariate analyses at the *P* < 0.2 level (Table 1).

## Results

### Patient characteristics

Table 1 shows the patients’ sociodemographic characteristics across all clinics/EDs (overall) and by the safety-net and private healthcare systems. Of the 564 patients surveyed, most were female (72%), Hispanic or Latinx (47%), African American or Black (33%), and visited safety-net clinics (72%). Most patients were insured through Medicaid or the county financial assistance program (57%) (Table 1).

Table 4 shows the patients’ composite scores for each situational summary factor. Overall, the accessibility of non-prescribed antibiotics was cited by more than half of the respondents as a situation predisposing influencing their intention to use non-prescription antibiotics (54%). Over half of the patients surveyed reported experiencing at least one situation related to previous symptom relief with antibiotics (53%). More than a third of patients reported barriers to a doctor’s visit (37%) (Table 4).

### Healthcare system differences

Table 2 shows the number and percentage of patients that endorsed intended use for the specific situations queried, grouped by the healthcare system. More patients expressed their intention to use non-prescribed antibiotics for each situation in the safety-net clinics than those seen in private EDs.

Many patients reported high intended use (51.3% for safety-net and 37.4% for private) for situations regarding symptom relief with antibiotics (summary factor 2) and accessibility of non-prescribed antibiotics (summary factor 3). For instance, “*you got better by taking the antibiotic before*” (public 51% vs private 37%; *P* = 0.012), “*your doctor prescribed you the antibiotic for the same symptoms before*” (public 53.8% vs private 38.7%; *P* < 0.001), and “*you have leftover antibiotics at home from a previous prescription*” (public 55.5% vs private 36.8%; *P* < 0.001) (Table 2). In addition, the situations related to summary factor 1, barriers to a doctor’s visit, were frequently reported across both healthcare systems. For example, “*you cannot take time off work*” (public 30.8% vs private 12.9%; *P* < 0.001), having “*no time to go to the doctor because of family/caregiving responsibilities*” (pubic 30.8% vs private 14.8%; *P* < 0.001), or “*a visit with a doctor is too expensive*” (public 35.5% vs private 20%; *P* = 0.007) increased patients’ intended use (Table 2).

### Multivariate linear regression models

Table 5 shows the multivariate linear regression results for the 3 situational summary factors. *Summary factor 1: Barriers to a doctor visit.* Compared to private ED patients, the patients receiving care through the safety-net clinics had endorsed higher intended use due to barriers to a doctor’s visit (β [standard error, SE] = 1.34 [0.24]; *P* < 0.001). Younger patient age was associated with higher intended use for this summary factor (β [SE] = −0.02 [0.008]; *P* = 0.031). Patient gender, race and ethnicity, education level, insurance status, language preference, and health literacy level were not significant predictors in this model (Table 5).

**Table 5.** Multivariable linear regression results for each situational summary factor outcome

	Intended use of non-prescription antibiotics by situational summary factor:					
	Summary factor 1: Barriers to a doctor’s visit		Summary factor 2: Accessibility of non-prescribed antibiotics		Summary factor 3: Previous symptom relief with antibiotics	
Sociodemographic factors^a^	β (std. error)	*P* value	β (std. error)	*P* value	β (std. error)	*P* value
Age	−0.02 (0.008)	*P* = 0.031	−0.016 (0.005)	*P* = 0.001	−0.01 (0.003)	*P* = 0.002
Healthcare system						
Public safety-net clinics (vs private EDs)	1.34 (0.24)	*P* < 0.001	0.75 (0.154)	*P* < 0.001	0.36 (0.096)	*P* < 0.001

a All models were adjusted for age, sex, race/ethnicity, education, insurance, healthcare system, survey language, and health literacy. Patient sex, race/ethnicity, education, insurance, survey language, and health literacy were insignificant in the models.

*Summary factor 2: Accessibility of non-prescribed antibiotics.* Compared to private ED patients, the patients receiving health care through the safety-net clinics had higher intended use because of the accessibility of non-prescribed antibiotic sources (β [SE] = 0.75 [0.154]; *P* < 0.001). Younger age was associated with more intended use due to the accessibility of non-prescribed antibiotic sources (β [SE] = −0.016 [0.005]; *P* = 0.001). Patient gender, race and ethnicity, education level, insurance status, language preference, and health literacy level were not significant predictors in this model (Table 5).

*Summary factor 3: Previous symptom relief with antibiotics.* Safety-net clinic patients had higher intended use than private ED patients because of prior symptom relief with antibiotics for similar/same symptoms (β [SE] = 0.36 [0.096]; *P* < 0.001). Younger age was associated with higher intended use due to previous symptom relief using antibiotics (β [SE] = −0.01 [0.003]; *P* = 0.002). Patient gender, race and ethnicity, education level, insurance status, language preference, and health literacy level were insignificant predictors in this model (Table 5).

## Discussion

Our study revealed alarmingly high proportions of patients endorsing intended non-prescription antibiotic use across all 13 predefined situations. Up to half (30–50%) of all patients surveyed expressed an intention to use antibiotics without a prescription for situations involving high doctor visit costs, possessing leftover prescription antibiotics, and having experienced prior symptom relief when using antibiotics. In addition, our adjusted results found that younger patient age and receiving care from the safety-net clinics was associated with increased intention to use non-prescription antibiotics across all summary factors (ie, perceived barriers to a doctor visit, accessibility of non-prescription antibiotics, and previous symptom relief with antibiotics).

The high percentage of patients intending to use leftover antibiotic courses (50.4%) or using antibiotics because their doctor had prescribed it previously (49.6%) highlights the ongoing, systemic problem of overprescribing. Clinicians often prescribe antibiotics for self-limiting symptoms that do not typically require antibiotic courses (eg, acute respiratory infections) or prescribe longer courses than recommended by guidelines.^21–23^ Some of this prescribing behavior is driven by perceived patient expectations of receiving antibiotics.^24^

Additionally, the large percentage of patients reporting prior symptom relief with antibiotic treatment (47.5%) indicates a substantiated need for additional education on appropriate antibiotic use for patients. Other studies show patients’ trust in primary care professionals’ clinical advice, including willingness to consider alternative/nonantibiotic treatment options if counseled appropriately and involved in shared decision-making regarding antibiotics.^25,26^ In addition, patient-provider counseling focused on the individual (patient-level) adverse outcomes (eg, *Clostridioides difficile* infection and severe drug interactions/complications) may resonate with patients and may have more individual relevance than discourse about societal harms or antibiotic resistance resulting from inappropriate antibiotic use.^5,27^

Our findings also highlight that younger patients report higher intentions to use non-prescription antibiotics for situations related to healthcare system barriers, accessibility of non-prescribed antibiotics, and beliefs that antibiotics will alleviate symptoms experienced. These findings are consistent with other European and US-based studies that also found younger patient age groups were more likely to use non-prescription antibiotics.^6,28–30^ Younger patients may have lower knowledge around antibiotics, resistance, and how to care for self-limiting infections than older patients. Additionally, younger patients may not have an established primary care doctor compared with older patients.

### Limitations

Despite having a large, diverse outpatient sample with representation across healthcare systems, including public primary care clinics and private EDs, our study findings may not be generalizable to all US-based patient populations. However, other large US cities with demographics similar to that of Houston may have similar results. For instance, the Rice University Kinder Institute projected that by 2040, most US cities will resemble the sociodemographics of the greater Houston metroplex.^31^ Additionally, our survey relied on self-reporting; thus, a social desirability response bias may have occurred despite our best efforts to phrase questions neutrally. Some patients may not disclose their antibiotic use without a prescription due to stigma, discrimination, or legalities surrounding the practice. Moreover, the coronavirus disease 2019 pandemic and quarantine may have introduced unintended contextual changes that may have impacted responses. Finally, because patients were surveyed in the healthcare setting, they may be more prone to seek healthcare advice, and these results may be an underrepresentation of the true burden of non-prescription antibiotic use. More research is needed to expand on these results and investigate these factors in other geographically, racially, ethnically, and linguistically diverse study populations in the United States.

Our results broadly align with the priorities of the United States Government Office of the Assistant Secretary for Planning and Evaluation (ASPE).^32^ The ASPE reports that patients’ social factors can negatively impact over 50% of their health outcomes.^32^ The ASPE encourages integrating patients’ social situations into public health, health care, social services, and other governmental institutions in collaboration to advance health equity and improve patient health outcomes and well-being.^32^ In addition, in a recent survey of over 1,500 US physicians, social factors adversely impacted patients’ health behaviors and outcomes.^33^ Approximately 77% of the surveyed physicians stated that all or many of their patients had reported at least 1 social determinant that adversely impacts their health care, with financial instability (34%) and transportation (24%) being the most frequently reported social factors, according to patients.^33^

## Conclusions

Awareness and recognition of patients’ situational circumstances may help healthcare practitioners and policymakers target the underlying reasons why patients intend to use non-prescription antibiotics in future interventions. Future policies and antibiotic stewardship interventions should consider improving patient access to health care and tailoring education focused on safe antibiotic use. Clinicians may choose to provide medically appropriate, alternative (nonantibiotic) treatment options and information to patients experiencing certain symptoms where antibiotic treatments may not be warranted.

## Supporting information

Laytner et al. supplementary material Laytner et al. supplementary material

## Supplementary material

For supplementary material accompanying this paper visit http://doi.org/10.1017/ash.2024.361. click here to view supplementary material

## References

[1] Blumenthal KG , Peter JG , Trubiano JA , Phillips EJ. Antibiotic allergy. Lancet 2019;393:183–198. 30558872 10.1016/S0140-6736(18)32218-9 PMC6563335

[2] Yang L , Bajinka O , Jarju PO , et al. The varying effects of antibiotics on gut microbiota. AMB Express 2021;11:116. 34398323 10.1186/s13568-021-01274-w PMC8368853

[3] Morgan DJ , Okeke IN , Laxminarayan R , et al. Non-prescription antimicrobial use worldwide: a systematic review. Lancet Infect Dis 2011;11:692–701. 21659004 10.1016/S1473-3099(11)70054-8 PMC3543997

[4] World Health Organization. Antimicrobial resistance. https://www.who.int/news-room/fact-sheets/detail/antimicrobial-resistance. Published 2023. Accessed September 13, 2022.

[5] Sun G , Manzanares K , Foley KA , et al. Antibiotic stewardship with upper respiratory tract infection patients at student health centers: providers’ communication experiences and strategies. Am J Infect Control 2023;51:154–158. 35605753 10.1016/j.ajic.2022.05.013

[6] Sun R , Yao T , Zhou X , et al. Non-biomedical factors affecting antibiotic use in the community: a mixed-methods systematic review and meta-analysis. Clin Microbiol Infect 2022;28:345–354. 34768017 10.1016/j.cmi.2021.10.017

[7] Grigoryan L , Haaijer-Ruskamp FM , Burgerhof JG , et al. Self-medication with antimicrobial drugs in Europe. Emerg Infect Dis 2006;12:452–459. 16704784 10.3201/eid1203.050992 PMC3291450

[8] Torres NF , Chibi B , Kuupiel D , et al. The use of non-prescribed antibiotics; prevalence estimates in low-and-middle-income countries. A systematic review and meta-analysis. Arch Public Health 2021;79:2. 33390176 10.1186/s13690-020-00517-9 PMC7780654

[9] Lescure D , Paget J , Schellevis F , van Dijk L. Determinants of self-medication with antibiotics in European and Anglo-Saxon countries: a systematic review of the literature. Front Public Health 2018;6:370. 30619809 10.3389/fpubh.2018.00370 PMC6304439

[10] Grigoryan L , Paasche-Orlow MK , Alquicira O , et al. Antibiotic use without a prescription: a multi-site survey of patient, health system, and encounter characteristics. Clin Infect Dis 2023;77:510–517. 37094252 10.1093/cid/ciad241

[11] Zoorob R , Grigoryan L , Nash S , Trautner BW. Nonprescription antimicrobial use in a primary care population in the United States. Antimicrob Agents Chemother 2016;60:5527–5532. 27401572 10.1128/AAC.00528-16 PMC4997852

[12] Essigmann HT , Aguilar DA , Perkison WB , et al. Epidemiology of antibiotic use and drivers of cross-border procurement in a Mexican American border community. Front Public Health 2022;10:832266. 35356027 10.3389/fpubh.2022.832266 PMC8960039

[13] Grigoryan L , Germanos G , Zoorob R , et al. Use of antibiotics without a prescription in the U.S. population: a scoping review. Ann Intern Med 2019;171:257–263. 31330541 10.7326/M19-0505

[14] Laytner L , Chen P , Nash S , et al. Perspectives on non-prescription antibiotic use among Hispanic patients in the Houston metroplex. J Am Board Fam Med 2023;36:390–404. 37127346 10.3122/jabfm.2022.220416R1 PMC10706826

[15] Mainous AG, 3rd, Diaz VA , Carnemolla M. Factors affecting Latino adults’ use of antibiotics for self-medication. J Am Board Fam Med 2008;21:128–134. 18343860 10.3122/jabfm.2008.02.070149

[16] Larson EL , Dilone J , Garcia M , Smolowitz J. Factors which influence Latino community members to self-prescribe antibiotics. Nurs Res 2006;55:94–102. 16601621 10.1097/00006199-200603000-00004

[17] Sanchez J. Self-medication practices among a sample of Latino migrant workers in South Florida. Front Public Health 2014;2:108. 25140297 10.3389/fpubh.2014.00108 PMC4121528

[18] Grigoryan L , Burgerhof JG , Degener JE , et al. Attitudes, beliefs and knowledge concerning antibiotic use and self-medication: a comparative European study. Pharmacoepidemiol Drug Saf 2007;16:1234–1243. 17879325 10.1002/pds.1479

[19] Chew LD , Bradley KA , Boyko EJ. Brief questions to identify patients with inadequate health literacy. Fam Med 2004;36:588–594. 15343421

[20] Chew LD , Griffin JM , Partin MR , et al. Validation of screening questions for limited health literacy in a large VA outpatient population. J Gen Intern Med 2008;23:561–566. 18335281 10.1007/s11606-008-0520-5 PMC2324160

[21] Grigoryan L , Zoorob R , Wang H , Trautner BW. Low concordance with guidelines for treatment of acute cystitis in primary care. Open Forum Infect Dis 2015;2:ofv159. 10.1093/ofid/ofv159 PMC4675917 26753168

[22] Fleming-Dutra KE , Hersh AL , Shapiro DJ , et al. Prevalence of inappropriate antibiotic prescriptions among US ambulatory care visits, 2010-2011. JAMA 2016;315:1864–1873. 27139059 10.1001/jama.2016.4151

[23] Hersh AL , King LM , Shapiro DJ , et al. Unnecessary antibiotic prescribing in US ambulatory care settings, 2010-2015. Clin Infect Dis 2021;72:133–137. 32484505 10.1093/cid/ciaa667 PMC9377284

[24] Boiko O , Gulliford MC , Burgess C. Revisiting patient expectations and experiences of antibiotics in an era of antimicrobial resistance: qualitative study. Health Expect 2020;23:1250–1258. 32666579 10.1111/hex.13102 PMC7696122

[25] Spicer JO , Roberts RM , Hicks LA. Perceptions of the benefits and risks of antibiotics among adult patients and parents with high antibiotic utilization. Open Forum Infect Dis 2020;7:ofaa544. 10.1093/ofid/ofaa544 PMC7731524 33335939

[26] Coxeter PD , Mar CD , Hoffmann TC. Parents’ expectations and experiences of antibiotics for acute respiratory infections in primary care. Ann Fam Med 2017;15:149–154. 28289114 10.1370/afm.2040 PMC5348232

[27] Miller BJ , Carson KA , Keller S. Educating patients on unnecessary antibiotics: personalizing potential harm aids patient understanding. J Am Board Fam Med 2020;33:969–977. 33219075 10.3122/jabfm.2020.06.200210 PMC7791407

[28] Petersen MR , Cosgrove SE , Quinn TC , et al. Prescription antibiotic use among the US population 1999–2018: National Health and Nutrition Examination Surveys. Open Forum Infect Dis 2021;8:ofab224. 10.1093/ofid/ofab224 PMC8291435 34295941

[29] Olmeda K , Trautner BW , Laytner L , et al. Prevalence and predictors of using antibiotics without a prescription in a pediatric population in the United States. Antibiotics (Basel) 2023;12:491. 36978358 10.3390/antibiotics12030491 PMC10044616

[30] Anderson A. Antibiotic self-medication and antibiotic resistance: multilevel regression analysis of repeat cross-sectional survey data in Europe. REGION 2021;8:121–145.

[31] Binkovitz L. Projections show how Houston, and the country, will change by 2030. https://kinder.rice.edu/urbanedge/projections-show-how-houston-and-country-will-change-2030. Published 2017.

[32] Whitman A , De Lew N , Chappel A , et al. Addressing Social Determinants of Health: Examples of Successful Evidence-based Strategies and Current Federal Efforts. Washington, D.C.: Assistant Secretary for Planning and Evaluation (ASPE); 2022.

[33] Physicians Foundation. 2022 survey of America’s physicians. https://physiciansfoundation.org/wp-content/uploads/2022/03/SDOH-Survey-Report.pdf. Published 2022. Accessed January 2, 2023.

