# Supplementary material for: Situations predisposing primary care patients to use antibiotics without a prescription in the United States
Source: Antimicrob Steward Healthc Epidemiol. 2024 Sep 9;4(1):e121. doi: 10.1017/ash.2024.361 (PMC11384165; doi:10.1017/ash.2024.361)
Supplement: Laytner et al. supplementary material [file S2732494X24003619sup001.docx]

**Appendix 1. Survey Questions**

Survey questions (to be asked and completed by research coordinators)

*Throughout this survey, I will often use the word antibiotic(s). An antibiotic is a medication that is used to fight infections. Please feel free to ask me to repeat or clarify what an antibiotic is at any point during this survey.*

*“The following set of questions will be over situations where you might use antibiotics.”*

1. If you were feeling sick, would you take antibiotics in the following situations without contacting a doctor/nurse/dentist/clinic?

*Check answer.*

|  | Yes | No | Don’t Know |
| --- | --- | --- | --- |
| You cannot take time off work. |  |  |  |
| You have no time to go to the doctor because of family responsibilities. |  |  |  |
| You cannot get to the doctor’s office because of transportation problems. |  |  |  |
| The doctor’s office hours are not convenient for you. |  |  |  |
| The doctor has no time to see you when you are sick. |  |  |  |
| A visit with a doctor is too expensive. |  |  |  |
| You got better by taking this antibiotic before. |  |  |  |
| Your doctor prescribed you this antibiotic for the same symptoms before. |  |  |  |
| Antibiotics are cheaper than over the counter cold and flu medications. |  |  |  |
| Friends/relatives give you antibiotics. |  |  |  |
| You can buy antibiotics without a prescription in the United States. |  |  |  |
| You can buy antibiotics without a prescription in another country.  If yes, please specify: |  |  |  |
| You have leftover antibiotics at home from a previous prescription. |  |  |  |

*“In the following section we will ask a little more about you.”*

1. How old are you? ____
2. Sex
   - Male
   - Female
   - Other: ___________
3. Do you consider yourself to be Hispanic/Latino?
   - Yes
   - No
4. Which category best describes your race?
   - Black or African American
   - White
   - Declined
   - Other, please specify: __________________________
5. What is the highest level of education you have completed?
   - Never attended school
   - Grades 1 through 5 (Elementary)
   - Grades 6 through 8 (Middle School)
   - Grades 9 through 11 (Some High School)
   - Grades 12 or GED (High School graduate)
   - College 1 year to 3 years (Some college or technical school)
   - College 4 years or more (College graduate)
6. What was the total annual income in your household in the past year?
   - Less than $20,000
   - $20,000 or more but less than $40,000
   - $40,000 or more but less than $60,000
   - $60,000 or more but less than $100,000
   - More than $100,000
   - Don’t know/prefer not to say
7. Which of the following health insurance plans do you have? (Mark all that apply)
   - Medicaid
   - Medicare
   - Harris Health System/Gold card
   - None
   - Other:________________________
8. Where were you born?
   - United States
   - Other, please specify: _________________________
9. How many years have you lived in the United States? ________

*“The following set of questions will be over your healthcare experiences.”*

1. How often do you have problems learning about your medical condition because of difficulty understanding written information?
   - Never *Go to Question #13*
   - Occasionally
   - Sometimes
   - Often *Continue to Question #12*
   - Always
2. Was this because the material was not written in your native language?
   - Yes
   - No
3. How confident are you filling out medical forms by yourself?
   - Extremely
   - Quite a bit
   - Somewhat
   - A little bit
   - Not at all
4. How often do you have someone help you read clinic or hospital materials?
   - Never
   - Occasionally
   - Sometimes
   - Often *Continue to Question #15*
   - Always
5. Was this because the material was not written in your native language?
   - Yes
   - No
